# Association of Alpha-1 Antitrypsin Pi*Z Allele Frequency and Progressive Liver Fibrosis in Two Chronic Hepatitis C Cohorts

**DOI:** 10.3390/jcm12010253

**Published:** 2022-12-29

**Authors:** Victoria Therese Mücke, Janett Fischer, Marcus Maximilian Mücke, Alexander Teumer, Alexander Koch, Johannes Vermehren, Malin Fromme, Stefan Zeuzem, Christian Trautwein, Christoph Sarrazin, Thomas Berg, Biaohuan Zhou, Karim Hamesch

**Affiliations:** 1 Department of Internal Medicine 1, University Hospital Frankfurt, Goethe University Frankfurt am Main, 60590 Frankfurt, Germany; 2 Division of Hepatology, Department of Medicine II, Leipzig University Medical Center, 04103 Leipzig, Germany; 3 Department of Psychiatry and Psychotherapy, University Medicine Greifswald, 17475 Greifswald, Germany; 4 Medical Clinic III, Gastroenterology, Metabolic Diseases and Intensive Care, University Hospital RWTH Aachen, 52074 Aachen, Germany; 5 Department of Gastroenterology, St. Josefs-Hospital, 65189 Wiesbaden, Germany; 6 Department of Surgical Oncology, Fujian Provincial Hospital, Fuzhou 350001, China

**Keywords:** alpha-1 antitrypsin deficiency, heterozygous Pi*Z carriage, liver fibrosis, hepatitis C virus, *SERPINA1*

## Abstract

(1) Background: The inherited alpha-1 antitrypsin (A1AT) deficiency variant ‘Pi*Z’ emerged as a genetic modifier of chronic liver disease. Controversial data exist on the relevance of heterozygous Pi*Z carriage (‘Pi*MZ’ genotype) as an additional risk factor in patients with chronic viral hepatitis C to develop progressive liver fibrosis. (2) Methods: Two prospectively recruited cohorts totaling 572 patients with therapy-naïve chronic viral hepatitis C (HCV) were analyzed. The Frankfurt cohort included 337 patients and a second cohort from Leipzig included 235 patients. The stage of liver fibrosis was assessed by liver biopsy, AST-to-platelet ratio index (APRI) score and Fibrosis-4 (FIB-4) score (Frankfurt) as well as liver stiffness measurement (LSM) via transient elastography (Leipzig). All patients were genotyped for the Pi*Z variant (rs28929474) of the *SERPINA1* gene. (3) Results: In the Frankfurt cohort, 16/337 (4.7%) patients carried the heterozygous Pi*Z allele while 10/235 (4.3%) in the Leipzig cohort were Pi*Z carriers. In both cohorts, there was no higher proportion of Pi*Z heterozygosity in patients with cirrhosis compared to patients without cirrhosis or patients with cirrhosis vs. no liver fibrosis. Accordingly, Pi*Z frequency was not different in histological or serological stages of liver fibrosis (F0–F4) and showed no clear association with LSM. (4) Conclusions: Evaluation in two representative HCV cohorts does not indicate Pi*Z heterozygosity as a clinically relevant disease modifier in chronic HCV infection. However, validation in even larger cohorts with longitudinal follow-up is warranted.

## 1. Introduction

More than 30 years after the first description of the hepatitis C virus (HCV) genome [1], chronic HCV infection—despite the introduction of direct acting antiviral therapy to cure the disease [2]—still represents a serious health problem affecting more than 184 million people worldwide [3]. However, the course and the spectrum of chronic HCV disease is highly variable and includes asymptomatic carriers as well as patients who develop progressive liver disease with liver fibrosis/cirrhosis and development of hepatocellular carcinoma. Multiple modifiable and non-modifiable factors associated with disease severity have been identified [4]. However, there are no reliable predictive methods that allow accurate estimation of HCV liver disease progression [5].

Among Europeans, α1-antitrypsin (A1AT) deficiency is one of the most common hereditary diseases causing, among others, lung emphysema and liver disease [6,7]. A1AT is an acute phase protein primarily produced within hepatocytes [8]. Over 100 mutations of the A1AT gene (*SERPINA1*) are described, while the most relevant mutation is called the “Pi*Z” variant (Gly342Lys substitution). Individuals carrying the Pi*Z variant may retain polymerized A1AT protein within their hepatocytes which leads to varying degrees of reduced serum concentrations of A1AT [9]. Homozygous carriers of the Pi*Z variant (“Pi*ZZ” genotype) may develop progressive liver disease [10,11,12,13,14,15]. Recently, a multinational cohort study revealed that heterozygous carriage of the Pi*Z variant (“Pi*MZ” genotype) is a strong disease modifier in metabolic liver disease (i.e., alcoholic and non-alcoholic fatty liver disease) [16,17,18]. The impact of heterozygous Pi*Z carriage is also controversially discussed in other chronic liver diseases [6,19,20]. Regarding chronic HCV infection, there have been controversial data on the impact of heterozygous Pi*Z carriage in the last decades, and some studies have reported an increased risk for liver fibrosis progression [21,22,23], while others have not [21,24,25].

In view of these contradictory findings, we aimed to evaluate the prevalence of heterozygous carriage of the Pi*Z variant in different stages of liver fibrosis and cirrhosis in two large and well-characterized cohorts of patients with chronic HCV infection.

## 2. Materials and Methods

A total of 572 patients with chronic HCV infection were prospectively recruited in two German tertiary centers, University of Frankfurt (*n* = 337) and University of Leipzig (*n* = 235). Patients were recruited before initiation of HCV treatment in the time period from 2006 to 2008. At that time, patients consented to genetic testing and analyses and the study protocol was approved by the responsible local ethics committee of participating centers. Main exclusion criteria in both cohorts were age <18 years, pregnancy and co-infection with human immune deficiency virus or hepatitis B virus. The patients in the Frankfurt cohort were originally included in a prospective HCV treatment study, excluding patients with previous antiviral therapy, patients with serum creatinine levels ≥1.5 mg/dL, platelets <80/nL and patients with decompensated liver disease. In addition, only patients with an average alcohol intake <40 g/d (female) and <60 g/day (male) were included. Patients’ characteristics for subgroups of these cohorts were described previously [26,27,28,29]. For this study, stored blood samples of these cohorts were used for retrospective analysis.

In both cohorts, the degree of liver fibrosis or cirrhosis was documented. In the first cohort (Frankfurt) all patients underwent liver biopsy. The presence of liver fibrosis was assessed by local pathologists according to internationally standardized criteria from F0 = no fibrosis detected to F4 = presence of cirrhosis according to the METAVIR score [30]. Additionally, the serologic fibrosis scores aspartate-transferase-to-platelet ratio index (APRI) and fibrosis-4 score (FIB-4) were calculated. APRI for patients infected with chronic hepatitis C were defined by the following cut-offs according to the existing literature assessing APRI in HCV-related liver disease: <0.5 ruled out significant liver fibrosis and >1 was consistent with advanced liver fibrosis/cirrhosis [31]. Respectively, fibrosis degrees determined by FIB-4 were defined as: <1.45 as F0–F1, >3.25 as F3–F4 fibrosis [32].

In the second cohort (Leipzig), liver biopsy results were available in 24 patients and 193 patients received vibration-controlled transient elastography (TE; FibroScan, Echosens, Paris, France) to determine liver stiffness measurement (LSM). All TE examinations were carried out by experienced physicians and standardized protocols. Two different LSM cut-off values were applied: cut-offs that were well-established for chronic HCV infection [33] (≥7.1 kPa: consistent with significant liver fibrosis (F ≥ 2); ≥9.5 kPa: consistent with advanced fibrosis (F ≥ 3); ≥12.5 kPa consistent with liver cirrhosis) as well as cut-offs that were used for A1AT-related liver disease (≥7.1 kPa: consistent with significant liver fibrosis (F ≥ 2); ≥10 kPa: consistent with advanced fibrosis (F ≥ 3); ≥13 kPa consistent with liver cirrhosis) [11,12,17,34]). In cases where both liver biopsy and TE values were available, biopsy results superseded LSM results categorizing stage of liver fibrosis. In 28 patients of the Leipzig cohort, diagnosis of cirrhosis was based on the combination of liver imaging (ultrasound or computer tomography imaging) and laboratory findings. FIB-4 score and APRI were assessed as described above.

Genomic DNA from all patients were genotyped for the presence of the Pi*Z variant (rs28929474, also known as p.E342K or Glu342Lys) of *SERPINA1* as described before [11].

Statistical calculations were performed using BiAS software version Windows 11.05.–12/2016 (epsilon-Verlag, Nordhastedt, Germany). Graphs were created with Prism for Windows (v5.02; GraphPad Software Inc., San Diego, CA, USA). Continuous variables were displayed as mean ± standard deviation (SD). Categorical variables were reported as absolute (*n*) and relative (%) frequencies. Group differences were assessed by the Mann–Whitney U test and Fisher’s exact test for continuous or categorical variables, respectively. All tests were two-sided and *p*-values < 0.05 were considered statistically significant. Mantel–Haenszel linear-by-linear test for trends was used to assess the relationship between advanced fibrosis stage and Pi*Z heterozygosity. Multivariable logistic regression models were used to test for independent prediction and odds ratios (ORs) were given with their corresponding 95% confidence intervals (CI) in brackets.

## 3. Results

### 3.1. Patients’ Characteristics

The total study population (*n* = 572) included 281 men (49.1%). Among all patients, 26 patients (4.5%) could be identified as heterozygous Pi*Z carriers (Pi*MZ).

The Frankfurt cohort consisted of 337 HCV positive patients, 16 of them (4.7%) were heterozygous for the Pi*Z variant. Pi*Z non-carriers and carriers did not differ in their demographic characteristics. Moreover, the distribution of the different HCV genotypes was comparable among both groups (Table 1).

The Leipzig cohort comprised 235 patients including 10 (4.3%) Pi*Z carriers. The mean age of the cohort was 53.6 ± 13.9 years with a mean body mass index (BMI) of 23.3 ± 6.4 kg/m^2^; 125 were male (45.5%). Again, Pi*Z non-carriers and carriers did not differ in their demographic characteristics. Further characteristics including diagnosis of diabetes mellitus and arterial hypertension are depicted in Table S1.

### 3.2. Frankfurt Cohort—No Difference in Pi*Z Frequencies in Patients with Different Stages of Liver Fibrosis

In terms of liver-related blood parameters, HCV-positive patients who also were heterozygous Pi*Z carriers had slightly higher serum gamma-glutamyltransferase (GGT) activities than their HCV-positive counterparts that did not carry the Pi*Z variant (91.4 U/L vs. 67.3 U/L, *p* = 0.046; Table 1). No differences in other liver-related blood parameters were detected among both groups (Table 1).

All 337 patients of the Frankfurt cohort received liver biopsies. As shown in Table 2, most patients presented with F1 fibrosis (*n* = 139, 41.2%), followed by F0 (*n* = 77, 22.8%) and F2 fibrosis (*n* = 73, 21.7%). None of the Pi*Z carriers had biopsy-proven cirrhosis (F4). We could not detect a significant difference in the distribution of Pi*Z carriers and non-carriers regarding each stage of fibrosis. Additionally, the serologic fibrosis parameters APRI and FIB-4 score did not significantly differ between Pi*Z carriers and non-carriers (*p* = 0.444 and *p* = 0.516, respectively, Table 2). Concordantly, we could not detect significant differences in Pi*Z carriers vs. non-carriers regarding APRI (*p* = 1.0) and FIB-4 (*p* = 1.0) determined fibrosis degrees using cut-off values as described above (Figure S1).

**Figure 1:**
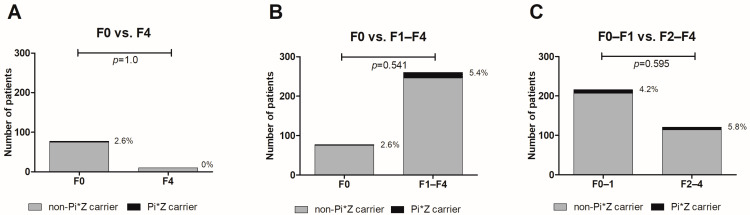
No difference in Pi*Z carrier frequencies in different stages of biopsy-proven liver fibrosis. Comparison of patients with F0 versus F4 fibrosis (**A**), F0 versus F1–4 fibrosis (**B**) and F0–F1 versus F2–4 fibrosis (**C**). Proportions of Pi*Z carriers (%) are depicted next to the bars.

The frequencies of Pi*Z carriers were comparable among patients without biopsy-proven fibrosis (stage F0) versus biopsy-proven cirrhosis (stage F4) (*p* = 1.0, Figure 1A). Similarly, we did not observe a significant difference in the prevalence of Pi*Z carriage in patients without biopsy-proven fibrosis (F0) versus any stage of fibrosis (F1–F4) (*p* = 0.541, Figure 1B). The same was true for the comparison of no/mild fibrosis (F0–1) versus fibrosis stage F2–F4 (*p* = 0.595, Figure 1C). In univariable and multivariable analyses, Pi*Z heterozygosity was not associated with no/lower biopsy-proven fibrosis stages (F0–F1) or advanced biopsy-proven stages of liver fibrosis (F3–F4). As expected, age (OR = 1.079, CI=1.038–1.122, *p* < 0.001) and BMI (OR = 1.107, CI = 1.021–1.200, *p* = 0.014) predicted advanced liver fibrosis (Table 3).

**Figure 2:**
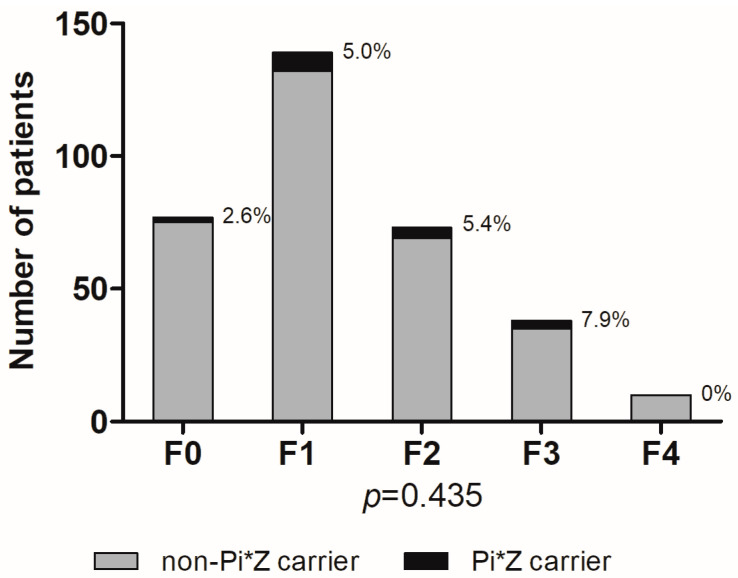
No trend in Pi*Z carrier frequencies in patients with chronic hepatitis C infection and different stages of biopsy-proven liver fibrosis (F0–F4). Proportions of Pi*Z carriers (%) are depicted next to the bars.

In addition, there was no significant trend for progressive liver fibrosis (F0–F4) in patients with Pi*Z heterozygosity (Figure 2).

### 3.3. Leipzig Cohort—No Difference in Pi*Z Frequencies in Patients with/without Cirrhosis and No Association with Liver Stiffness or Serologic Fibrosis Parameters

**Figure 3:**
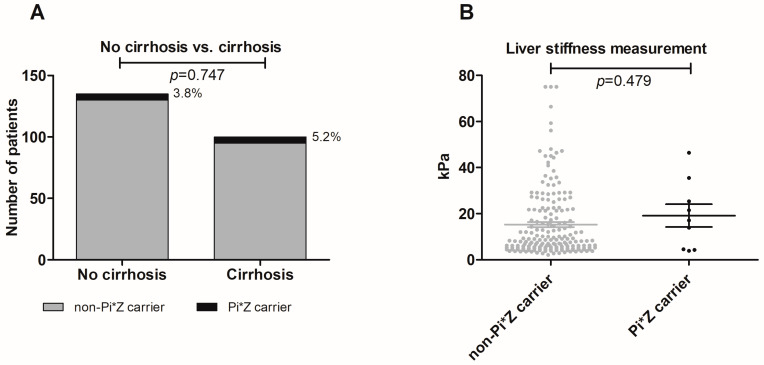
No difference in Pi*Z carrier frequencies in patients with chronic hepatitis C infection with/without cirrhosis (**A**). No association of Pi*MZ status and liver stiffness levels (determined by transient elastography) in patients with chronic hepatitis C infection (**B**). Proportions of Pi*Z carriers (%) are depicted next to the bars.

A second cohort of chronic HCV-infected patients from Leipzig was analyzed to validate our results: 100 (42.6%) patients of this cohort had liver cirrhosis. Here, five HCV patients with cirrhosis could be identified as Pi*Z carriers (2.1%). However, there was no significant difference between prevalence of Pi*Z heterozygosity comparing patients with versus without cirrhosis (*p* = 0.747; Figure 3A). In multivariable analysis, only age (OR = 1.037, CI = 1.013–1.062, *p* = 0.003) and BMI (OR = 1.046, CI = 1.023–1.069, *p* < 0.001) independently influenced presence of cirrhosis but not sex, HCV genotype 3, diabetes mellitus, arterial hypertension or the Pi*Z status (Table S2).

Similar to the Frankfurt cohort, APRI and FIB-4 score did not significantly differ between Pi*Z carriers and non-carriers (*p* = 0.705 and *p* = 0.355, respectively, Table 3). Moreover, there were no significant differences in Pi*Z carriers vs. non-carriers regarding APRI- and FIB-4-determined fibrosis degrees using cut-off values as described above in the Frankfurt cohort (*p* = 0.675 and *p* = 0.483, respectively; Figure S2).

Mean liver stiffness determined by TE examination was 15.5 ± 14.9 kPa (n = 193). We analyzed whether the A1AT genotype Pi*MZ correlated with LSM in HCV patients. As shown in Figure 3B, there was no significant difference between both groups (*p* = 0.479). If patients were divided into groups regarding stage of liver fibrosis determined by LSM cut-off values as described above, neither cut-off values established for patients with HCV nor cut-off values established for patients with A1AT deficiency could differentiate between Pi*Z carriers and non-carriers (Table 4).

## 4. Discussion

The current investigation evaluated the association of heterozygous Pi*Z carriage in different stages of liver fibrosis (F0–F4) in patients chronically infected with HCV. This study included a total of 572 prospectively recruited patients made up of two series of HCV-infected patients from two tertiary care centers, being the largest number of HCV-infected patients analyzed on this matter. In these HCV-infected patients there was no significant association between the Pi*MZ genotype and the stage of liver fibrosis.

For many years, the role of heterozygous Pi*Z carriage in the pathogenesis of chronic liver disease has been a matter of debate. Some initial studies suggested heterozygous A1AT deficiency as an important co-factor in the progression of chronic liver disease [21,22,35,36], while others did not [21,37,38]. A recent prospective multinational study showed the additive impact of heterozygous Pi*Z carriage on liver disease progression in patients with alcoholic or non-alcoholic fatty liver disease (ALD/NAFLD) [16] This strong association was confirmed in another multicentric cohort of ALD and NAFLD [39]. However, analyses in patients with underlying chronic HCV infection were either underrepresented or controversary so far: In the 1990s, Eigenbrodt et al. and Graziadei et al. evaluated the prevalence of abnormal A1AT phenotypes in small subgroups of patients with chronic HCV and end-stage liver disease who were considered for liver transplantation, reporting an OR of 4.3 and 4.6, respectively, of having a heterozygous Pi*Z phenotype [35,40]. In the same decade, Serfaty et al. made opposing observations in a case control study of 84 hospitalized HCV patients in which the heterozygous Pi*MZ genotype was no risk factor for cirrhosis [25]. Previous studies addressing this issue were very heterogenous in the methodology and consisted of small and non-representative numbers of patients with little data on potential biases such as additive alcohol intake, co-infections or metabolic factors. Moreover, previous studies had no information on anti-HCV therapeutic status, and the distribution of HCV genotypes was unknown. This is, however, important, as HCV genotype three is described to be associated with accelerated liver fibrosis progression [41].

More recent data were also contradictory: Regev et al. conducted a case-control study of patients with/without liver disease, including a small subgroup of HCV-infected patients, where they identified a higher prevalence of heterozygous Pi*Z state in the group of patients with decompensated liver disease than in the group with less severe liver disease [23]. Motawi et al. compared three groups of HCV-infected patients, asymptomatic HCV carriers (*n* = 100), chronic hepatitis patients (*n* = 85) and cirrhotic patients (*n* = 65). Interestingly, they found the Pi*MZ genotype mostly in patients with chronic hepatitis (5.9%), followed by the group of HCV carriers (5.0%), but none in the group of HCV cirrhotic patients (0%) [21]. Scott et al. distinguished between HCV patients with no fibrosis, intermediate fibrosis and cirrhosis in a total of 141 patients [21,24]. Their data left no hints that inherited heterozygous Pi*Z carriage influenced the severity of liver fibrosis.

In our much larger cohort of HCV-infected patients, we observed a slightly higher frequency of Pi*MZ genotypes (4.5%) compared to the prevalence of the normal population in Germany (1.9–4%) [42,43]. The population at hand consisted of 54.9% patients with HCV genotype three and there was no difference of HCV genotype distribution among Pi*Z carriers vs. non-carriers. However, our results did not reveal an association of Pi*Z carriage and the prevalence of cirrhosis or a significant higher degree of liver fibrosis: neither in a well-characterized characterized cohort using liver biopsy and serum-based fibrosis tests (Frankfurt), nor in our second cohort characterized by TE assessment and serologic parameters (Leipzig). Taken together with multiple reports in smaller cohorts [21,24,25], the Pi*Z variant does not seem to have a major or clinically meaningful impact on HCV-induced liver fibrogenesis. Hence, routine assessment for A1AT deficiency using serum A1AT levels of patients with solely HCV-related liver disease seems less promising to identify patients at risk of developing progressive liver injury. However, this interpretation has to be seen with caution as all published studies on this research question have a comparably low statistical power. 

The first limitation of our study is that despite of being numerically the hitherto largest cohort of HCV-infected patients analyzed on this matter, the absolute number of included Pi*Z carriers is still relatively small (26 of 572 patients), and despite analyzing two representative and well-characterized cohorts, the presented data cannot fully exclude an impact of Pi*MZ genotype on HCV-induced liver fibrogenesis. While greater powered cross-sectional studies would help reducing the risk of false-negative associations, longitudinal analyses are completely missing. A longitudinal analysis in non-treated HCV patients would help to decipher the role of the Pi*Z variant on the natural history of hepatitis C progression. However, presumably there are not likely to be many future studies on the natural history of untreated HCV infection since the invention and success of direct acting antivirals fundamentally changed the clinical management as viral eradication is possible in >95% of patients across different populations. Additionally, invasive assessment of underlying liver fibrosis in HCV-infected patients is no longer essential part of the typical work up [44], and hence it is unlikely that there will be larger cohorts of therapy-naïve HCV patients with biopsy-proven fibrosis staging in the future. Secondly, as a result of the study’s retrospective design, patients’ characterizing data is limited and possibly prone to reporting and information bias. However, all patients were part of two prospectively established cohorts at the time with clear inclusion criteria and are comparatively well characterized and provide more information on co-factors than previous reports. However, more information on further co-factors or longitudinal data would have been desirable. Thirdly, we partly used different modalities to evaluate the stage of liver fibrosis as only a few patients in the Leipzig cohort underwent liver biopsy. Nevertheless, the majority received non-invasive liver stiffness measurements via transient elastography, which has already been validated to adequately classify liver fibrosis [45]. In addition, we also took APRI and FIB-4 into account in both cohorts. Fourthly, we used liver biopsy, liver stiffness measurements and indirect non-invasive fibrosis tests (i.e., APRI and FIB-4). However, using direct non-invasive fibrosis tests (e.g., pro-C3 or enhanced liver fibrosis test (ELF)) might be a valuable addition to further cross-validate our findings. Taken together, despite these significant limitations, these two cohorts still resemble the most representative investigation of the Pi*Z variant frequency in HCV-related liver fibrosis so far and, thus, add pertinent knowledge to this open research question.

In conclusion, the systematic evaluation in two representative and well-characterized cohorts using liver biochemistry, liver elastography and liver biopsy did not show a relevant association of heterozygous carriage of the Pi*Z variant with signs of HCV-associated liver fibrosis. While the presented results have to be interpreted with caution due to the relatively small number of Pi*MZ patients with therapy-naïve HCV infection, these analyses might help to estimate the disease-modifying impact of the Pi*Z variant on progression of HCV-related liver disease. Data from larger HCV cohorts with longitudinal follow-up evaluating the impact of the Pi*Z variant on the natural history of HCV infection and regression after DAA therapy are desirable.

## Figures and Tables

**Table 1:** Patients’ characteristics in the Frankfurt cohort.

Parameters	Total, *n* = 337	Pi*Z Non-Carriers, *n* = 321	Pi*Z Carriers, *n* = 16	Significance (*p* Value)
Age (years)	42.7 ± 11.1	42.6 ± 11.2	45.6 ± 8.2	0.228
Men (*n*)	156 (46.2)	146 (45.5)	10 (62.5)	0.207
BMI (kg/m²)	25.7 ± 4.5	25.6 ± 4.5	26.9 ± 3.6	0.087
HCV genotype				0.756
1 (*n*)	60 (17.8)	56 (17.4)	4 (25)	
2 (*n*)	86 (25.5)	83 (25.9)	3 (18.8)	
3 (*n*)	185 (54.9)	176 (54.8)	9 (56.3)	
5 (*n*)	6 (1.8)	6 (1.9)	0 (0)	
ALT (U/L)	81.6 ± 58.7	81.5 ± 58.5	86.2 ± 64.2	0.879
AST (U/L)	56.0 ± 39.1	55.9 ± 39.1	56.9 ± 40.5	0.864
GGT (U/L)	68.5 ± 70.9	67.3 ± 70.6	91.4 ± 75.2	0.046 *
Bilirubin (mg/dL)	0.7 ± 0.3	0.7 ± 0.3	0.6 ± 0.3	0.117
Albumin (g/dL)	4.5 ± 0.4	4.4 ± 0.3	4.5 ± 0.3	0.248
Creatinine (mg/dL)	0.8 ± 0.2	0.8 ± 0.2	0.8 ± 0.1	0.388
Hemoglobin (g/dL)	14.8 ± 1.4	14.8 ± 1.4	14.7 ± 1.4	0.607
Leucocytes (/nL)	6.9 ± 2.1	6.9 ± 2.1	7.2 ± 1.9	0.338
Platelets (/nL)	240 ± 63	240 ± 63	244 ± 71	0.959
HbA1c (%)	5.4 ± 0.4	5.4 ± 0.4	5.3 ± 0.3	0.378
HOMA-IR score	3.2 ± 3.4	3.2 ± 3.5	2.4 ± 2.0	0.528

Abbreviations: BMI, body mass index; HCV, hepatitis C virus; ALT, alanine transferase; AST, aspartate transferase; GGT, gamma-glutamyltransferase; HbA1c, hemoglobin A1c; HOMA-IR, homeostasis model assessment of insulin resistance; * *p* < 0.05. Missing data: bilirubin *n* = 3, albumin *n* = 11, hemoglobin *n* = 1, leucocytes *n* = 1, platelets *n* = 1, HbA1c *n* = 57, HOMA-index *n* = 53.

**Table 2:** Fibrosis parameters of the Frankfurt cohort.

Parameters	Total, *n* = 337	Pi*Z Non-Carriers, *n* = 321	Pi*Z Carriers, *n* = 16	Significance (*p* Value)
Biopsy-determined stage of fibrosis				
F0 (*n*)	77 (22.8)	75 (23.4)	2 (12.5)	0.541
F1 (*n*)	139 (41.2)	132 (41.1)	7 (43.6)	1.0
F2 (*n*)	73 (21.7)	69 (21.5)	4 (25)	0.753
F3 (*n*)	38 (11.2)	35 (10.9)	3 (18.6)	0.405
F4 (*n*)	10 (3.0)	10 (3.1)	0 (0)	1.0
Serologic scores				
APRI score	0.56 ± 0.21	0.56 ± 0.21	0.52 ± 0.21	0.444
APRI score < 0.5 (*n*)	152 (45.1)	143 (44.5)	9 (56.2)	0.443
APRI score > 1 (*n*)	8 (2.3)	8 (2.5)	0 (0)	1.0
FIB-4 score	1.27 ± 1.01	1.28 ± 1.03	1.21 ± 0.55	0.516
FIB-4 score < 1.45 (*n*)	248 (73.6)	235 (73.2)	13 (81.3)	0.575
FIB-4 Score > 3.25 (*n*)	15 (4.5)	15 (4.7)	0 (0)	1.0

Abbreviations: APRI, aspartate transferase to platelet ratio index; FIB-4, fibrosis-4 score. Missing data: APRI *n* = 1, FIB-4 *n* = 1. APRI fibrosis degree: <0.5 = ruled out, >1 = associated with cirrhosis; FIB-4 fibrosis degree: <1.45 = F0–F1, >3.25 = F3–F4.

**Table 3:** Multivariable analysis of the binary outcome no/mild liver fibrosis (F0-F1) versus advanced liver fibrosis (F3–F4).

Variables (*n* = 216)	Univariable Analysis		Multivariable Analysis	
	OR (95% CI)	*p* Value	OR (95% CI)	*p* Value
Age	1.080 (1.041–1.121)	<0.001	1.079 (1.038–1.122)	<0.001
Female sex	1.532 (0.725–3.324)	0.264		
Body mass index	1.113 (1.033–1.198)	0.005	1.107 (1.021–1.200)	0.014
Genotype 3	1.061 (0.506–2.228)	0.875		
Hemoglobin A1c	1.831 (0.864–3.879)	0.114		
Pi*Z heterozygosity	2.330 (0.569–9.550)	0.240		

**Table 4:** Fibrosis parameters of the Leipzig cohort.

Parameters	Total, *n* = 235	Pi*Z Non-Carriers, *n* = 225	Pi*Z Carriers, *n* = 10	Significance (*p* Value)
**LSM (kPa)**	15.5 ± 14.9	15.3 ± 15.0	19.2 ± 14.8	0.479
LSM cut-offs HCV, (*n*)				
<7.1 kPa	80 (34.0)	77 (34.2)	3 (30)	1.0
7.1–<9.5 kPa	24 (10.2)	24 (10.7)	0 (0)	0.604
9.5–<12.5 kPa	13 (5.5)	13 (5.8)	0 (0)	1.0
≥12.5kPa	76 (32.3)	70 (31.1)	6 (60)	0.081
LSM cut-offs A1ATD, (*n*)				
<7.1 kPa	80 (34.0)	77 (34.2)	3 (30)	1.0
7.1–<10.0 kPa	25 (10.6)	25 (11.1)	0 (0)	0.605
10.0–<13.0 kPa	14 (6.0)	14 (6.2)	0 (0)	1.0
≥13.0 kPa	74 (14.8)	68 (30.2)	6 (60)	0.076
**Serologic scores**				
APRI score	1.85 ± 2.05	1.86 ± 2.09	1.63 ± 1.12	0.705
APRI score < 0.5 (*n*)	44 (18.7)	43 (19.1)	1 (10)	0.693
APRI score > 1 (*n*)	118 (50.2)	112 (49.8)	6 (60)	0.749
FIB-4 score	4.24 ± 4.01	4.20 ± 4.03	5.01 ± 3.63	0.355
FIB-4 score < 1.45 (*n*)	59 (25.1)	57 (25.3)	2 (20)	1.0
FIB-4 Score > 3.25 (*n*)	92 (39.1)	86 (38.2)	6 (60)	0.195

Abbreviations: LSM, liver stiffness measurement; HCV, hepatitis C virus; A1ATD, alpha-1 antitrypsin deficiency; APRI, aspartate transferase to platelet ratio index; FIB-4, fibrosis-4 score. Missing data: Age *n* = 2, BMI *n* = 81; LSM *n* = 42; APRI *n* = 15; FIB-4 *n* = 17. APRI fibrosis degree: <0.5 = ruled out, >1 = associated with cirrhosis; FIB-4 fibrosis degree: <1.45 = F0–F1, >3.25 = F3–F4.

## Data Availability

The data that support the findings of this study are available on request from the corresponding author, K.H.

## Supplementary Materials

The following supporting information can be downloaded at: https://www.mdpi.com/article/10.3390/jcm12010253/s1, Figure S1: No difference in Pi*Z carrier frequencies in different stages of serologically defined liver fibrosis; Figure S2: No difference in Pi*Z carrier frequencies in different stages of serologically defined liver fibrosis; Table S1: Patients’ characteristics of the Leipzig cohort; Table S2: Multivariable analysis of the binary outcome liver cirrhosis and no liver cirrhosis of the Leipzig cohort.
