# Supplementary material for: Association of Alpha-1 Antitrypsin Pi*Z Allele Frequency and Progressive Liver Fibrosis in Two Chronic Hepatitis C Cohorts
Source: J Clin Med. 2022 Dec 29;12(1):253. doi: 10.3390/jcm12010253 (PMC9821389; doi:10.3390/jcm12010253)

Supporting information

Mücke et al.: Association of alpha-1 antitrypsin allele Pi*Z frequency and progressive liver fibrosis in two chronic hepatitis C cohorts

Table S1. Patients' characteristics of the Leipzig cohort.

**Parameters**	**Total, *n*=235**	**Pi*Z non-carriers, *n*=225**	**Pi*Z carriers, *n*=10**	**Significance (*p* value)**
Age (years)	53.6±13.9	53.7±14.0	53.4±12.6	0.874
Men (***n***)	125 (45.5)	108 (48)	7 (70)	0.208
BMI (kg/m²)	23.3±6.4	23.0±6.1	31.9±10.7	0.053
ALT (U/l)	76.4±58.7	77.9±59.5	45.1±20.8	0.107
AST (U/l)	75.6±54.7	76.5±55.6	55.8±19.4	0.490
GGT (U/l)	110.5±172.8	110.9±175.6	101.6±85.8	0.524
Bilirubin (mg/dl)	16.5±12.8	16.6±12.9	13.4±9.3	0.354
Albumin (g/dl)	3.6±0.8	3.6±0.8	3.5±0.5	0.449
Platelets (/nl)	169±81	171±80	146±91	0.284
**HCV genotype 3**	25 (10.6)	23 (10.2)	2 (20)	0.288
**Diabetes mellitus**	46 (19.6)	44 (8.6)	2 (20)	1.0
**Arterial hypertension**	89 (37.8)	86 (38.2)	3 (30)	0.746

Abbreviations: BMI, body mass index; HCV, hepatitis C virus, ALT, alanine transferase; AST, aspartate transferase; GGT, gamma glutamyl-transferase. Missing data: age *n*=2; BMI *n*=30, ALT *n*=13, AST *n*=12, GGT *n*=15, bilirubin *n*=24, albumin *n*=34, platelets *n*=14, HCV genotype n=13.

Table S2. Multivariable analysis of the binary outcome liver cirrhosis and no liver cirrhosis of the Leipzig cohort.

**Variables (*n*=219)**	**Univariable analysis**		**Multivariable analysis**	
	**OR (95% CI)**	***p* value**	**OR (95% CI)**	***p* value**
Age	1.040 (1.016-1.064)	<0.001	1.037 (1.013-1.062)	0.003
Female sex	0.529 (0.299-0.937)	0.029		
Body mass index	1.047 (1.025-1.069)	<0.001	1.046 (1.023-1.069)	<0.001

Genotype 3	1.329 (0.549-3.242)	0.532
Diabetes mellitus	2.156 (1.054-4.411)	0.035
Arterial hypertension	2.150 (1.200-3.851)	0.010
Pi*Z heterozygosity	1.646 (0.426-6.367)	0.470

Figure S1. No difference in Pi*Z carrier frequencies in different stages of serologically defined liver fibrosis. Comparison of HCV patients of the Frankfurt cohort with aspartate transferase to platelet ratio index (APRI) <0.5 versus >1 (**A**) and fibrosis-4 score (FIB-4) <1.45 versus >3.25 (**B**). Proportions of Pi*Z carriers (%) are depicted next to the bars.

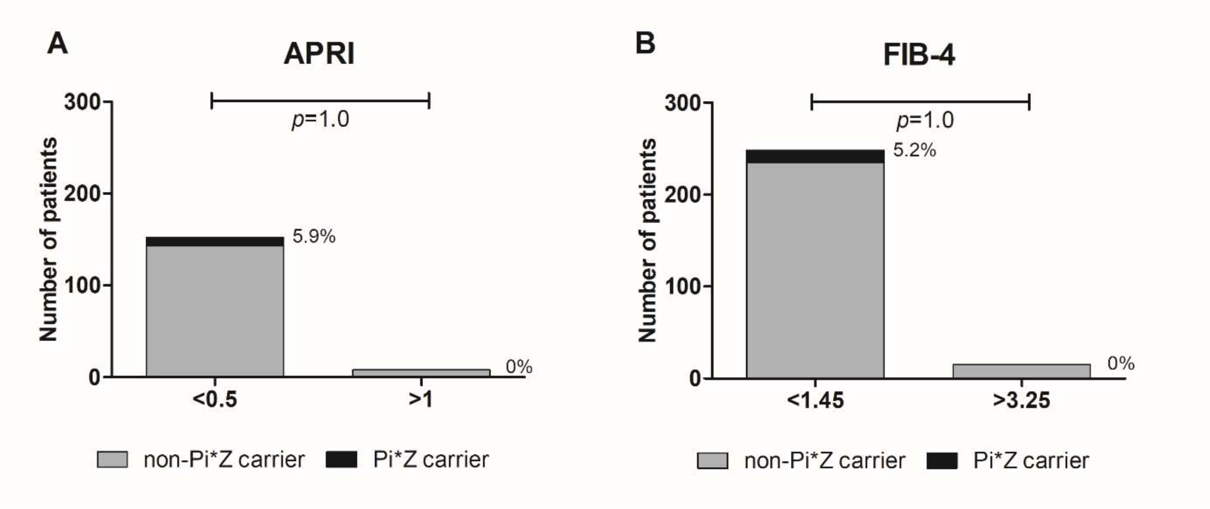

Figure S2. No difference in Pi*Z carrier frequencies in different stages of serologically defined liver fibrosis. Comparison of HCV patients of the Leipzig cohort with aspartate transferase to platelet ratio index (APRI) <0.5 versus >1 (**A**) and fibrosis-4 score (FIB-4) <1.45 versus >3.25 (**B**). Proportions of Pi*Z carriers (%) are depicted next to the bars.

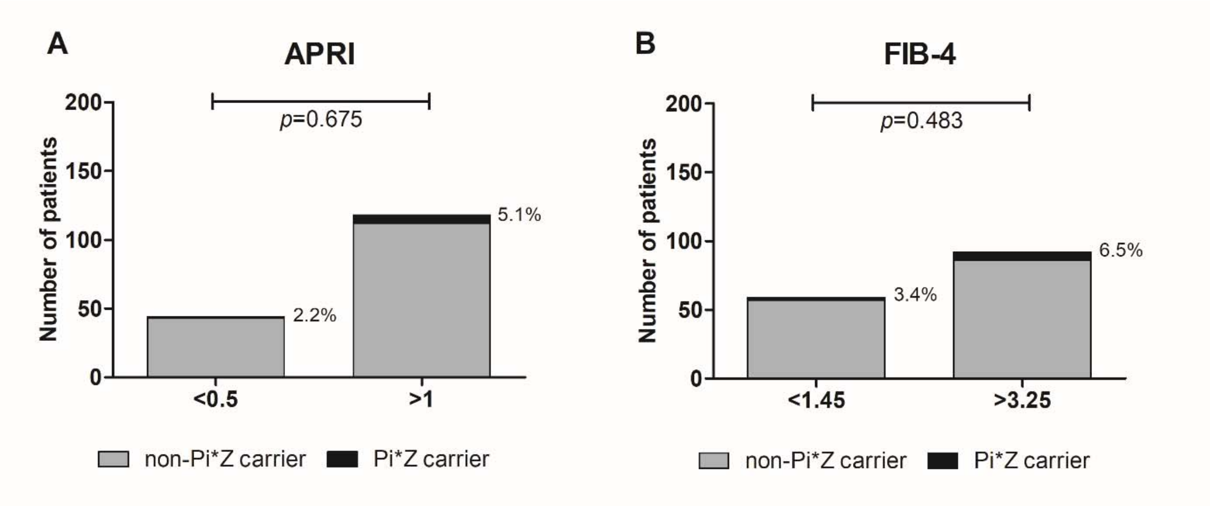

Supplement: Supplementary file 1 [file jcm-12-00253-s001.zip › jcm-2094079-SI.pdf]
